# Inflammation of the Mastoid Process

**Published:** 1871-04

**Authors:** 


Inflammation of the Mastoid Process, complicating inflammation of the middle ear, Prof. Roosa does not think receives sufficient attention from the profession. Such an affection is a very serious disorder, and its early diagnosis becomes a matter of much moment; a delay in this allows the extension of the disease to the brain through the foramina that transmit the branches of the meningial artery. He is in the habit, whenever there is deep-seated pain referred to the cavity of the tympanum, which is not relieved promptly by leeching and the warm douche, of regarding the case as an inflammation of the mastoid, and of making free incision into the parts, through the integument and periosteum. Experience shows that there is no danger in this procedure, while its neglect is often fatal. He resorts to the operation whenever there is pain, or tenderness, or swelling in the mastoid itself; especially when there is any reason to suspect caries of the bone, should an explorative incision be made. After such an incision, and the bone is found to be diseased, it should be perforated; this should be done when there is good reason to think there is pus in the mastoid cells which cannot find exit elsewhere. The mastoid should be perforated in case of supuration of long standing with frequent and painful exacerbations.—*Medical Record.*

